# Alterations in the serum metabolome in patients with the COVID-19 Omicron variant and in recovered cases

**DOI:** 10.1371/journal.pone.0327297

**Published:** 2025-10-14

**Authors:** Feng Gao, Daming Wang, Lihua Zuo, Junyi Sun, Bowen Dong, Ranran Sun, Yingying Shi, Ying Sun, Yawen Zou, Qingquan Jia, Na Han, Haiyu Wang, Liwen Liu, Guizhen Zhang, Zujiang Yu, Zhi Sun, Zhigang Ren

**Affiliations:** 1 Department of Infectious Diseases, State Key Laboratory of Antiviral Drugs, Pingyuan Laboratory, The First Affiliated Hospital of Zhengzhou University, Zhengzhou, China; 2 Department of Pharmacy, The First Affiliated Hospital of Zhengzhou University, Zhengzhou, China; 3 Department of Translational Medicine Center, The First Affiliated Hospital of Zhengzhou University, Zhengzhou, China; Kirklareli Universitesi, TÜRKIYE

## Abstract

Corona Virus Disease (COVID-19) has become a global public health crisis, and the Omicron variant has rapidly taken over as soon as it was detected Serum circulating metabolites can provide extensive insights into the pathogenesis and diagnosis of many diseases. We included 336 omicron variant cases (OC), 216 recovered cases (RC), and 380 healthy controls (HC) for untargeted metabolomics analysis and analyzed their serum metabolic profiles by liquid chromatography-tandem mass spectrometry. Principal component analysis, orthogonal partial least squares discriminant analysis, t-test analysis and false discovery rate were used to characterize the serum metabolites of OC and RC. In addition, a noninvasive diagnostic model for OC was developed using Receiver operating characteristic analysis. Finally, a correlation analysis was performed using data from our published articles. The results showed that compared with HC, five metabolites, including DL-stachydrine, D-(+)-pipecolinic acid, furazolidone, L-arginine and 5α-dihydrotestosterone glucuronide were significantly elevated and one metabolite, prenylcysteine, was significantly decreased in the serum of OC, and that the increase in L-arginine and the decrease in prenylcysteine led to impaired urea cycling and a high risk of developing atherosclerosis, respectively. These metabolites were not fully restored to healthy human levels in recovered cases. In addition, we constructed a noninvasive diagnostic model for distinguishing Omicron variant patients from healthy individuals based on the six differential metabolites, and achieved high diagnostic efficacy in both the discovery and validation cohorts. Finally, the results of the correlation analysis showed a strong correlation between the alterations in the oropharyngeal microbiome and serum metabolome and the clinical indicators in the omicron variant cases. This study was the first to characterize serum metabolites in OC and RC based on a large clinical cohort, and successfully constructed and validated a noninvasive diagnostic model for Omicron variant patients.

## Introduction

Coronavirus disease 2019 is caused by severe acute respiratory syndrome coronavirus 2 (SARS-CoV-2) and has spread rapidly since the first case was identified in late December 2019 in Wuhan, Hubei Province, China, and was declared a pandemic by the World Health Organization (WHO) in March 2020 [1]. According to the WHO, as of July 2023, the number of infected people worldwide has reached more than 600 million, and the cumulative number of deaths has exceeded 6 million. In the past few years, due to continuous mutations in the genes of coronaviruses, several mutant strains have emerged, including Alpha, Beta, Gamma, Delta, Epsilon and Omicron. Omicron variant is a new severely mutated SARS-CoV-2 variant called B.1.1.529. It was first identified in Botswana, South Africa and was quickly classified as a variant of concern (VOC) by the WHO upon discovery (VOC: Refers to a variety of worrisome variants that result in increased transmissibility, more severe disease progression, poorer treatment efficacy or detection failures) [2–4]. The Omicron variant is the most severely mutated of all currently known SARS-CoV-2 variants and carries mutations that would result in enhanced viral transmissibility and resistance to vaccine-induced immunity [5]. Studies have shown that the Omicron variant is less pathogenic and causes milder clinical symptoms than the ancestral SARS-CoV-2 due to its attenuated replication and its less fusogenic nature [6–8]. However, the Omicron variant has a very powerful ability to transmit due to its apparent immune evasion to existing vaccines [9,10]. This means that the existing COVID-19 vaccine is far from being protective in the population. Therefore, we must apply new tools for early detection of potentially asymptomatic infections to more effectively control the transmission of Omicron variant.

Omicron variant strains are more likely to attack the upper respiratory tract and less likely to cause infections in the lungs [11]. Therefore, the most common symptoms of Omicron variant infection are runny nose, headache, fatigue, sneezing and sore throat [12]. While only 50% of patients with the Omicron variant experience the classic three symptoms of fever, cough and loss of sense of smell and taste [13]. Clinical evidence also suggests that the Omicron variant is less pathogenic [14]. However, it has also been shown that the Omicron variant attacks the liver, heart, muscles and gastrointestinal tract of the host [15,16]. Nevertheless, it is unclear whether the reduced pathogenicity of Omicron variant infection is associated with the metabolic state of the host compared to previous variant strains.

Numerous studies have been conducted to show that microorganisms have an important role in the diagnosis and development of diseases [17–20]. However, the role of metabolites in disease is not well understood. Metabolites are an important component of human serum and abnormalities in serum metabolites are often observed in the early stages and during the progression of disease. Previous studies have shown that many viral infections, including SARS-CoV-2, cause dramatic changes in human serum metabolites [21,22]. For example, Páez-Franco *et al.* found that three alpha-hydroxy acids in COVID-19 patients increased with disease progression and correlated with lung injury and altered oxygen saturation levels [23]. However, the characteristics of the serum metabolites in Omicron variant patients remain unclear. Therefore, we performed a metabolomic analysis of serum samples from patients and recovered individuals with Omicron variant and compared them with healthy controls. Our results show that serum metabolites of Omicron variant patients are significantly different from those of healthy individuals, while serum metabolites of recovered individuals, although showing a trend of recovery, are still not fully normalized. In addition, we constructed a noninvasive diagnostic model by analyzing serum differential metabolites in Omicron variant patients and used a validation cohort to demonstrate the validity of serum metabolites as a diagnostic tool for Omicron infection.

## Materials and methods

### Study design

The design of this study followed the principles of prospective specimen collection and retrospective blinded evaluation, guided by the Declaration of Helsinki and the Rules of Good Clinical Practice. All confirmed cases and healthy controls signed a written informed consent before sample collection. The project was approved by the Ethics Committee of the First Affiliated Hospital of Zhengzhou University (L2021-Y429-002).

We prospectively collected serum samples from some hospitalized patients in January to February 2022 at the COVID-19 designated hospital in Henan Province. All patients were diagnosed with Omicron strain infection. The diagnosis is based on the “COVID-19 diagnosis and treatment Program Trial V.9 Guidelines” issued by the National Health Commission of the People’s Republic of China. After rigorous screening, we included 349 confirmed cases of the COVID-19 Omicron variant and 400 healthy controls. All patients were treated according to standard guidelines. We included serum samples from 230 omicron patients with confirmed recovery when the diagnosed patient met the discharge criteria in the diagnostic and treatment guidelines. Finally, all serum samples were tested and analyzed by untargeted metabolomics. Healthy controls were volunteers from the Physical Examination Center of the First Affiliated Hospital of Zhengzhou University.

### Ethics approval and consent to participate

The design of this study was based on the principles of prospective sample collection and retrospective blind evaluation, and performed in accordance with the Helsinki Declaration and Rules of Good Clinical Practice. This study was approved by the Institutional Review Board of the First Affiliated Hospital of Zhengzhou University (L2021-Y429-002). All participants signed a written informed consent form.

### Collection and pre-processing of serum samples

To ensure sufficient effective sample size, two sets of serum samples were collected from each subject. In order to avoid the influence of diet, drugs and other factors on serum metabolites, we asked each subject to fast for at least 9 hours. The patient’s venous blood was collected by a specially trained operator using a negative pressure blood collection tube and temporarily stored in an inert separator gel procoagulant tube. After standing at room temperature for 30 min, the blood was centrifuged in a centrifuge for 10 min (3000 rpm, 4°C), and the upper serum layer was transferred to an ep tube and stored in a refrigerator at −80°C.

In a biosafety cabinet, 800 μL of extraction solution (methanol: acetonitrile = 1:1) was added to 200 μL of serum sample, mixed thoroughly with shaking and put to −40°C for 2h to precipitate. Then the precipitated sample was centrifuged at 12000 rpm for 15 min at 4°C, and finally 100 μL of supernatant was taken for analysis. The Quality control samples (QC samples) were a mixture of 24 serum samples (10 μL each) extracted with 960 μL of extraction solution and the rest of the conditions were the same as for the serum samples.

### QC samples preparation

QC samples analysis is performed during the collection of metabolomics data from all samples, which can ensure the reliability of experimental results. Monitor the pressure change before and after each injection and the change in the retention time of the main peak of the total ion flow graph. After the instrument was stabilized, sample analysis was started. A QC sample was inserted every 10 samples to verify that the instrument was stable. Insert a solvent-only blank sample after each QC sample to avoid cross-contamination.

### LC-MS analysis and data acquisition

Chromatographic separation was performed on a Dionex Ultimate 3000 liquid chromatograph (Thermo Scientific, San Jose, USA). The chromatographic separation was performed on an ACQUITY UPLC BEH C18 column (100 mm*2.1 mm, 1.7 μm, Waters, USA) at a column temperature of 40°C and a flow rate of 0.3 mL/min, in which the A mobile phase consisted of water and 0.1% formic acid and the B mobile phase was acetonitrile. The metabolites were eluted using the following gradient: 0–1.0 min, 5% B; 1.0–5.5 min, 5–60% B; 5.5–7.5 min, 60–68% B; 7.5–9.0 min, 68–100% B; 9.0–11.5 min, 100% B. The volume of each sample was 5 μL.

The chromatograph was interfaced with Q-Exactive Orbitrap mass spectrometry (Thermo Fisher Scientific, San Jose, USA). The instrument scanned the primary ion mass range of 80 ~ 1200 m/z. The ion source temperature was 300°C and the ion transfer tube temperature was 320°C. The collision energies were 20, 40, and 60 eV, and the spray voltages were +3.5 or −2.8 kV, respectively. The mass spectra were obtained by using the full MS/ddms2 scan patterns operated in the positive and negative ion modes. The experimental samples were taken randomly. Mixed standard solutions were analyzed in the same method as biological samples.

Mass spectrograms and spectral data were recorded using Xcalibur software. Metabolites were extracted from raw data files using Compound Discoverer 2.1 software. The resulting comprehensive peak list (molecular weight, peak area and retention time) was exported for subsequent statistical analysis and metabolomics visualization.

### Statistics

The principal component analysis (PCA) and orthogonal projection to latent structure discriminant analysis model (OPLS-DA) was analyzed through “MetaboAnalyst” website (https://www.metaboanalyst.ca/). The OPLS-DA model was validated by repeating the permutation test 200 times. Using VIP values to assess correlation between metabolite and sample categorization. Statistical significance was analyzed using the Mann-Whitney U test, with P < 0.05 indicating a statistically significant difference. P-values for multiple tests were corrected by false discovery rate (FDR) using the Benjamini-Hochberg method. Receiver operating characteristic (ROC) analysis was used to diagnose Omicron variant patient samples and healthy controls. ROC curves were used to evaluate the diagnostic efficacy of potential biomarkers. It was performed using SPSS v.26.0 for Windows (SPSS, Chicago, Illinois, USA).

## Results

### Study design and participant characteristics

**Fig 1:**
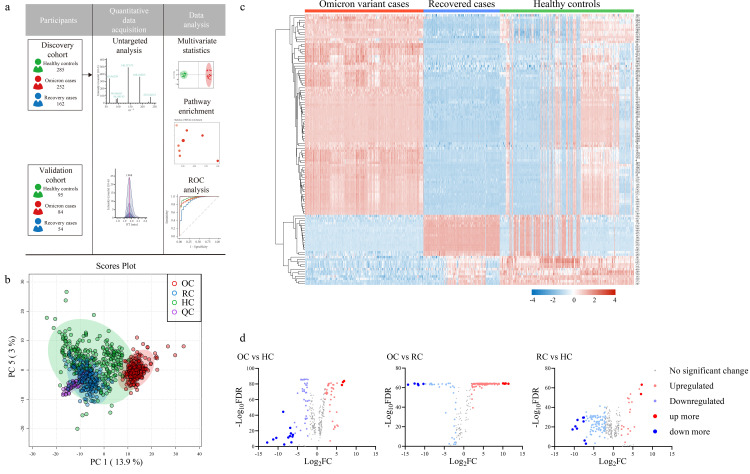
Untargeted metabolomic profiling of sera from Omicron variant cases of the discovery cohort. Untargeted metabolomic profiling using sera from HC, OC, and RC. **a)** Patients and study design; **b)** principal component analysis (PCA) of untargeted metabolomics among the three groups; **c)** Heat maps generated by 108 metabolites with VIP > 1 and FDR < 0.01 in orthogonal partial least squares discriminant analysis; **d)** volcano plots highlighted the serum metabolites that were increased (red) or decreased (blue) in the OC versus HC, OC versus RC and RC versus HC, with FDR < 0.01, log2 FC > 2 or <−2, VIP > 1. QC: quality control; OC, Omicron variant cases; RC, recovered cases; HC, healthy controls; FC, fold change; VIP, variable importance on projection; FDR, false discovery rate.

We prospectively collected 979 serum samples in Henan Province and subjected them to untargeted metabolomics (S1 Fig.). After a rigorous screening and exclusion process, a total of 932 serum samples were involved in the subsequent analysis. Among them, 336 Omicron variant cases (OC) samples, 216 recovered cases (RC) samples and 380 healthy controls (HC) samples were randomized into discovery cohort (252 OC, 162 RC and 285 HC) and validation cohort (84 OC, 54 RC and 95 HC). Serum metabolite profiles were then analyzed, serum metabolite profiles were described in a discovery cohort, metabolites with significant differences were identified and the best differential metabolites was identified as diagnostic markers, and a diagnostic model of the SARS-CoV-2 Omicron variety was constructed. In the validation cohort, we used 84 OC samples and 95 HC samples to validate the effectiveness of the diagnostic model (Fig 1a).

The clinical characteristics of the Omicron variant patients and healthy controls in the discovery cohort are shown in Table 1. The mean age of the Omicron variant patients was 27.49 years, with a male/female ratio of 137/115, while the mean age of the healthy controls was 23.32 years, with a male/female ratio of 175/110. 69 (27.4%) of the patients with the Omicron variant had symptoms of fever and 160 (63.5%) had symptoms of cough. We also analyzed the laboratory parameters of OC and HC. The results showed that compared to HC, OC had significantly lower leukocytes (p < 0.001), neutrophils (p < 0.001) and platelets (p < 0.001), but an increase of alanine aminotransferase (p < 0.005), total bilirubin (p < 0.001) and serum creatinine (p < 0.001), which initially indicated that Omicron variant patients had developed immune system dysfunction and abnormal liver and kidney function.

**Table 1:** Clinical characteristics of COVID-19 cases and healthy controls in the discovery cohort.

	COVID-19 cases (n = 252)	Healthy controls (n = 285)	p-value
**Age (years)**	27.49 ± 16.19	23.32 ± 1.27	<0.001
**Sex (female/male)**	137/115	175/110	0.099
**Comorbidities**	22 (8.7%)		
**Clinical signs and symptoms**			
** Fever**	69 (27.4%)		
** Cough**	160 (63.5%)		
**Rhinorrhoea**	25 (9.9%)		
** Myalgia**	1 (0.4%)		
** Fatigue**	11 (4.4%)		
** Headache**	6 (2.4%)		
** Diarrhoea**	3(1.2%)		
**Laboratory results**			
** Leukocytes (×10**^**9**^ **cells·L**^**-1**^**; NR 3.5–9.5)**	5.06 ± 1.45	6.09 ± 1.44	<0.001
** Neutrophils (×10**^**9**^ **cells·L**^**-1**^**; NR 1.8–6.3)**	2.61 ± 1.16	3.57 ± 1.13	<0.001
** Lymphocytes (×10**^**9**^ **cells·L**^**-1**^**; NR 1.1–3.2)**	1.92 ± 0.62	2.03 ± 0.55	0.026
** Platelets (×10**^**9**^ **cells·L**^**-1**^**; NR 125.0–350.0)**	206.23 ± 47.18	256.87 ± 52.03	<0.001
** Haemoglobin (g·L** ^**-1**^ **; NR 130.0–175.0)**	148.81 ± 33.76	140.71 ± 18.50	0.001
** ALT (U·L** ^**-1**^ **; NR 9.0–50.0)**	22.75 ± 20.03	17.68 ± 11.58	0.001
** AST (U·L** ^**-1**^ **; NR 15.0–40.0)**	20.92 ± 12.10	19.68 ± 7.95	0.915
** Total bilirubin (μmol·L** ^**-1**^ **; NR 0.0–21.0)**	12.72 ± 5.65	11.13 ± 6.19	<0.001
** Scr (μmol·L** ^**-1**^ **; NR 57.0–111.0)**	72.49 ± 12.84	66.14 ± 15.32	<0.001

Continuous variables are presented as the means (SD). ALT: alanine aminotransferase; AST: aspartate aminotransferase; Scr: serum creatinine. Categorical variables are presented as percentages. Differences between subjects with COVID-19 cases (n = 252) and healthy controls (n = 285) were compared by using Student’s t- test for normal continuous variables, the Wilcoxon rank- sum test for non- normal continuous variables and the χ2 test or Fisher’s exact test for categorical variables. Comorbidities included cardiovascular disease, diabetes, high blood pressure and chronic liver disease.

### Untargeted metabolomic profiling of sera of Omicron variant patients

**Fig 2:**
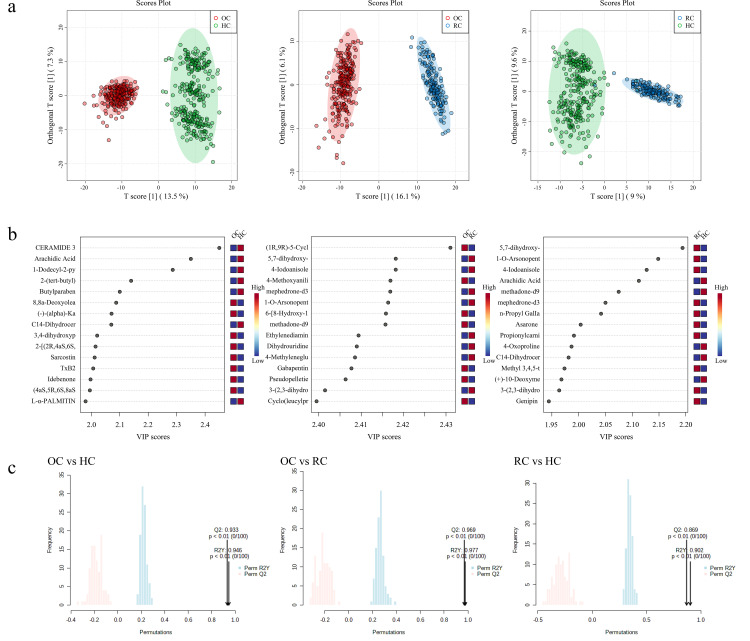
The orthogonal projection to latent structure discriminant analysis (OPLS-DA) analysis of the three groups (OC vs HC, OC vs RC and RC vs HC) of the discovery cohort. **a)** OPLS-DA showed the best possible discrimination of metabolites among OC, RC and HC as indicated. The x-axis represents the prediction component that shows differences between groups, and the y-axis represents the orthogonal component differences within the group; **b)** The 15 metabolites with the highest VIP values in the three groups; **c)** 100 permutation tests for three groups of OPLS-DA. R2 represents goodness of fit, Q2 represents goodness of prediction, and P value shows the significance level of the model.

We processed and detected all serum samples for non-targeted metabolomics data using liquid chromatography-mass spectrometry (LC-MS) technology according to a standardized protocol. Hydrophilic and hydrophobic molecules were analyzed in positive and negative ion modes, respectively, to cover various classes of circulating metabolites. We first used a self-constructed database for metabolite identification. For metabolites for which we did not have reliable standards in our database, we searched public databases using MS/MS spectra to improve the confidence of metabolite identification. PCA was used to differentiate metabolomic profiles between the groups of Omicron variant cases, recovered cases, and healthy controls. OPLS-DA was used for pairwise comparisons between three metabolites (Fig 2a). The permutation test was repeated 200 times to verify the reliability of the model and prevent overfitting (Fig 2c).

A total of 508 metabolites in the positive ion mode and 329 metabolites in the negative ion mode were finally identified (Data S1-S3). And significant changes in serum metabolites in Omicron variant cases compared to recovered cases and healthy controls (Fig 1b). The data from the quality control (QC) group showed consistency and reproducibility in the principal component analysis (PCA) (Fig 1b). Notably, the metabolites of RC were partially consistent with HC, while OC were easily distinguished from the other two groups (Figs 1b and 2a). 108 out of 837 metabolites were significantly associated with COVID-19 {Variable importance in the projection (VIP) >1.5, FDR < 0.01}. Based on the heatmap, it can be seen that the metabolites of RC and HC have high similarity and can be easily distinguished from OC (Fig 1c). Compared to the normal group, the volcano plot showed that OC had a significant increase in 40 metabolites and a decrease in 50 metabolites, while RC had an increase in 19 metabolites and a decrease in 97 metabolites. This number is even more striking when comparing the differences in circulating metabolites between OC and RC, with an increase of 106 and a decrease of 37 (Fig 1d and Data S4; VIP > 1.0, FDR < 0.01, log_2_FC > 2 or <−2). This illustrates that although patients in recovery met the discharge criteria, many of their metabolites did not return to normal levels compared to healthy individuals. It indicates that these discharged patients did not fully recover from the physiological effects of COVID-19.

### Pathway enrichment analysis of major differential metabolites

**Fig 3:**
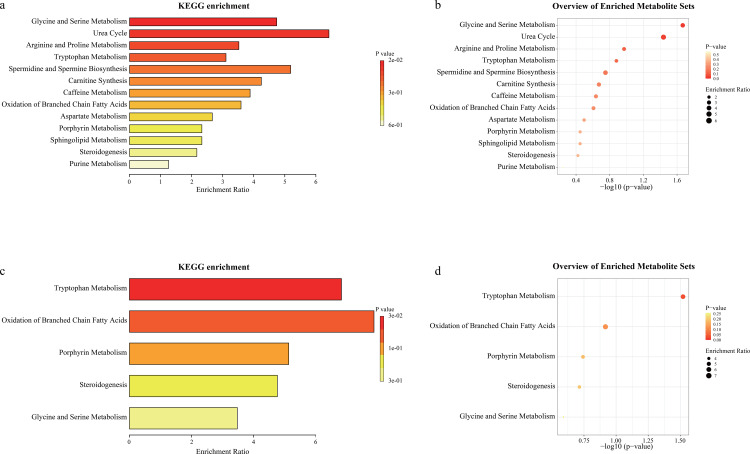
Metabolome KEGG enrichment analysis of serum in Omicron variant cases and recovered cases. **a, b)** KEGG enrichment analysis of differential metabolites (VIP > 1.0) in the group of Omicron variant cases. The color of the bubbles represents the adjusted p-value and the size of the bubbles represents the enrichment ratio; **c, d)** KEGG enrichment analysis of differential metabolites still present in the recovered cases.

To further analyze the metabolomics data, we performed Kyoto Encyclopedia of Genes and Genomes (KEGG) functional enrichment analysis for significantly differential metabolites (VIP > 1.0, after secondary matching) of OC compared to HC and the differential metabolites still present in RC, respectively, to reveal the potential functional implications of the differential metabolites in these groups and the extent of recovery of metabolic levels in RC (Fig 3). In the group of Patients with Omicron variant, differential metabolites were enriched to 13 metabolic pathways, and the three most important pathways included urea cycle, Spermidine and Spermine Biosynthesis and Glycine and Serine Metabolism (Figs 3a and b; Data S5). The urea cycle is carried out in the human liver, and its main function is to synthesize harmful ammonia in the body into harmless urea for excretion, so the abnormal urea cycle reflects to a certain extent that Patients with Omicron variant have different degrees of liver damage. The glycine and serine metabolism also takes place in the liver, and they are both eventually metabolized by the liver into energy substances to provide the body with energy. Abnormalities in this pathway not only reflect impaired liver function in Omicron variant patients, but also explain why they feel weak. And abnormal spermidine and spermidine metabolism may lead to reduced immune function in humans. In addition to these three most important pathways, carnitine synthesis, caffeine metabolism, oxidation of branched chain fatty acids and porphyrin metabolism are also abnormal to varying degrees in Patients with Omicron variant.

As for recovered patients with Omicron variant, differential metabolites were enriched in only 5 pathways and most metabolic pathways have returned to normal human levels (Figs 3c and d; Data S6). However, Glycine and Serine Metabolism, oxidation of branched chain fatty acids and porphyrin metabolism were still at abnormal levels. This suggests that although recovered individuals have met the criteria for discharge, their metabolic status has not fully returned to normal, and they likely still have an underlying inflammatory response and immune dysregulation.

### Significantly altered metabolites in Omicron variant patients

**Fig 4:**
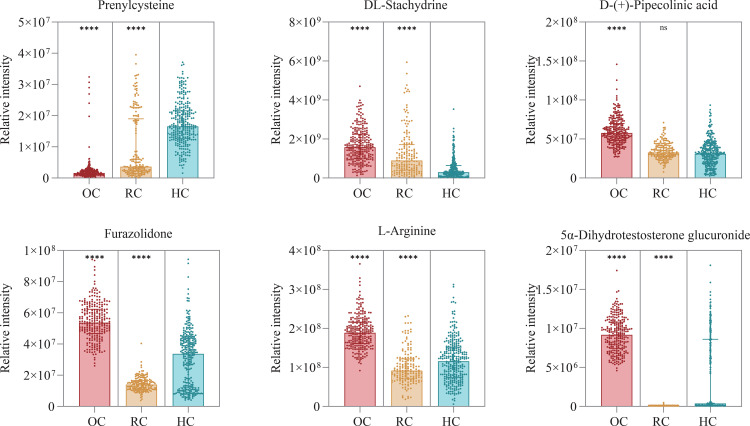
Relative intensities of differential metabolites that may serve as potential metabolic biomarkers in Omicron variant cases. Each group is represented by a different color, and each point represents a patient sample, as shown in the figure. OC, Omicron variant cases; RC, recovered cases; HC, healthy controls. ****P < 0.0001; ns: no significance.

By comparing serum metabolites from Omicron variant cases, recovered cases and healthy controls, we highlighted six different metabolites that were up- or down-regulated in Patients with Omicron variant. The five metabolites that were significantly upregulated were DL-stachydrine, D-(+)-pipecolinic acid, furazolidone, L-arginine and 5α-dihydrotestosterone glucuronide, while the one metabolite that were significantly downregulated were prenylcysteine (Fig 4 and Data S7).

These metabolites have an essential role in human physiological function. For example, L-arginine is an important intermediate in the urea cycle, which can be catalyzed by arginase to produce urea, thereby converting the highly toxic ammonia produced by protein metabolism in the body into urea for excretion. L-arginine was significantly upregulated in the Omicron variant cases, which we believe may be a result of impaired liver function leading to impaired urea circulation and ultimately to the accumulation of L-arginine. Prenylcysteine can react with it as a substrate for prenylcysteine oxidase 1 to produce oxidant substances, which produce atherogenic effects. Therefore, a decrease in prenylcysteine may increase the risk of late atherosclerosis in cases of Omicron variants. Notably, as patients with the Omicron variant recover, the relative levels of D-(+)-pipecolinic acid, furazolidone, L-arginine and 5α-dihydrotestosterone glucuronide have largely returned to normal, and prenylcysteine, DL-stachydrine and furazolidone, although still at dysregulated levels, have tended to converge like healthy individuals. This also reflects that the recovered cases have not fully recovered their metabolic levels from the effects of the Omicron strain, although they have met the discharge criteria.

### Linear regression and receiver operating characteristic curve analysis

To exclude the possibility of age differences leading to potential biomarkers, we developed a unary linear regression model for Patients with Omicron variant and healthy controls. All six significantly different differential metabolites had P values > 0.05 and R^2^ values close to 0, indicating that there was no significant association between age and metabolites (Data S8). Therefore, by analysis of unary linear regression between Patients with Omicron variant and healthy controls, we concluded that the effect of age on serum metabolites was not significant.

**Fig 5:**
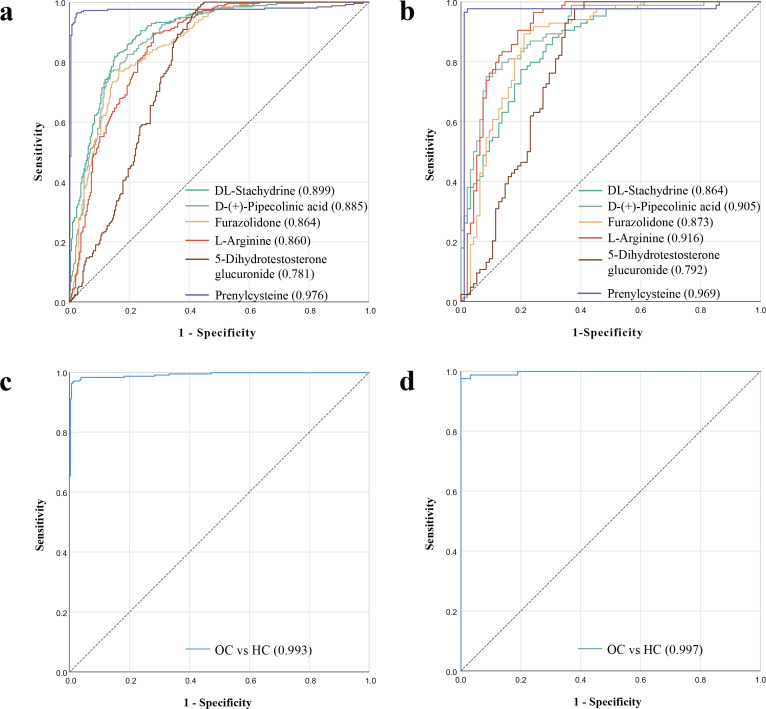
ROC curve analysis for the predictive power of biomarkers for distinguishing between patients with Omicron variant and healthy controls. **a)** In the discovery cohort, ROC curve analysis of the predictive power of five upregulated serum metabolites and one downregulated serum metabolite for distinguishing Omicron variant patients from healthy controls; **b)** Efficacy of 5 upregulated serum differential metabolites and one downregulated serum metabolite for the diagnosis of patients with Omicron variant in a validation cohort; **c)** In the discovery cohort, ROC curve analysis of the predictive power of combined 6 differential serum metabolites for distinguishing Omicron variant group from healthy controls; **d)** Efficacy of 6 differential serum metabolites in the validation cohort for the combined diagnosis of patients with Omicron variant. OC, Omicron variant cases; HC, healthy controls.

We then evaluated the potential efficacy of these serum differential metabolites for the diagnosis of Omicron variant cases by generating ROC curves. We analyzed the ROC curves for the five upregulated differential metabolites between Omicron variant cases and healthy controls, and results showed that DL-Stachydrine in the discovery cohort had the largest area under the curve (AUC) value of 0.899, while L-Arginine in the validation cohort had the largest AUC value of 0.916 (Figs 5a and b). We also analyzed the ROC curves for a down-regulated differential metabolite, prenylcysteine, and showed that the AUC values for prenylcysteine in the discovery cohort and validation cohort were 0.976 and 0.969, respectively (Figs 5a and b). By further ROC analysis, we found that the combined 6 serum differential metabolites could effectively differentiate between Omicron variant patients and healthy individuals, with AUC values of 0.993 and 0.997 for the discovery cohort and validation cohort, respectively (Figs 5c and d). This suggests that combining the six serum differential metabolites can serve as an effective noninvasive diagnostic marker for patients with the Omicron variant strain.

### Correlation analysis of microbiome, metabolome and clinical indicators in Omicron variant cases

**Fig 6:**
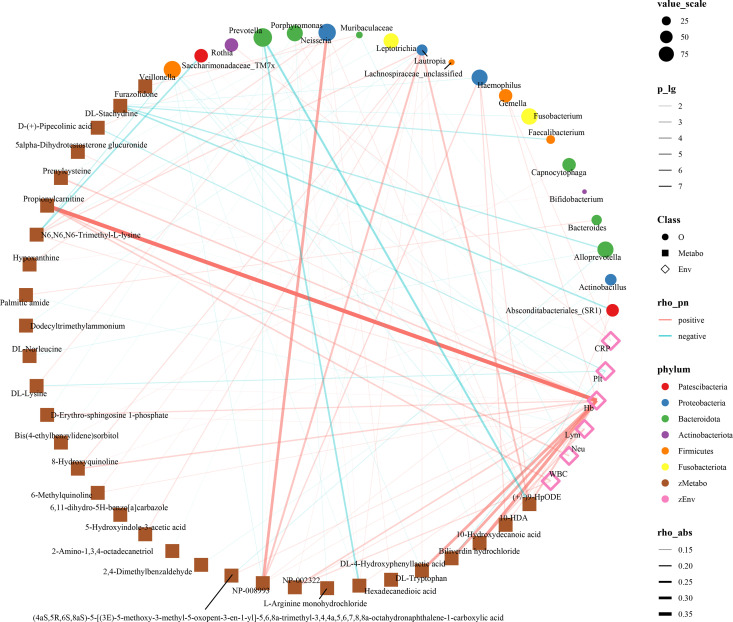
Analysis of the correlation between pharyngeal microbiomics, metabolomics and clinical indicators between OC and HC. We included microbiomes and metabolites that were significantly different in the OC for Spearman’s correlation analysis. The results showed correlations between 20 oral microbiomes, 31 serum metabolites, and 6 clinical indicators. The size of the dots indicates the relative abundance of genera, and the color of the dots indicates the different phylums of the microbiome. The transparency of the line indicates the negative logarithm of the correlation p-value (-lg P), the width of the line indicates the strength of the correlation (Spearman), the red line indicates positive correlation and the blue line indicates negative correlation. Circles represent the oral microbiome, squares represent serum metabolites, and diamonds represent clinical indicators.

The human microbiome and metabolites are inextricably linked in the development of disease. In another research, we found that the oropharyngeal microbiome of infected individuals with Omicron variant of COVID-19 was significantly different from that of healthy people, and that the oropharyngeal microbiome gradually returned to normal as the disease recovered [24]. Therefore, we analyzed the correlation between microbiome, metabolome, and clinical indicators in patients with Omicron variant. We included significantly different microbiomes and serum metabolites in OC for Spearman’s correlation analysis. The results showed correlations between 20 oral microbiomes, 31 serum metabolites, and 6 clinical indicators (Fig 6 and Data S9). Leukocytes were positively correlated with propionylcarnitine, and negatively correlated with oropharyngeal microbiome *Veillonella*. Lymphocytes were positively correlated with serum metabolites D-Erythro-sphingosine 1-phosphate and 10-Hydroxydecanoic acid, and negatively correlated with palmitic amide. Interestingly, hemoglobin was positively correlated with up to 16 (e.g., propionylcarnitine, 10-Hydroxydecanoic acid, biliverdin hydrochloride) serum metabolites and negatively correlated with only one oropharyngeal microorganism, *Veillonella*. In conclusion, these results suggest a close correlation between alterations in the oropharyngeal microbiome and serum metabolome and clinical indicators in Omicron variant cases.

## Discussion

A growing body of research suggests that metabolomics can be used as a non-invasive diagnostic tool in the early stages of many diseases [25–28]. This study provides the first systematic and comprehensive view of the serum metabolic profile of Omicron variant cases and recovered cases based on a large clinical cohort. Serum from Omicron variant cases and recovered cases was analyzed by untargeted metabolomics and compared with healthy individuals, and serum metabolites from Omicron variant cases were found to be significantly different from those of recovered cases and healthy individuals. We then identified potential biomarkers for disease diagnosis that distinguish Omicron variant cases from the healthy individuals. These will help us develop better strategies to diagnose and treat the disease.

L-arginine is an important intermediate metabolite in the urea cycle involved in the removal of excess ammonia from the body, and it participates in the final step of the urea cycle thereby converting harmful ammonia into urea for excretion from the kidneys [29,30]. We found that L-arginine levels were significantly increased in patients with the Omicron variant compared to normal levels, and L-arginine accumulation is usually associated with urea cycle disorders, suggesting that infection with the Omicron variant may lead to hepatic impairment in patients. We also demonstrated an abnormal urea cycle in patients with the Omicron variant by KEGG pathway enrichment analysis. Similar to our study, the study by Wu *et al*. found that the level of carbamoyl phosphate, an intermediate metabolite of the urea cycle, was significantly down-regulated in patients with COVID-19 and correlated with the severity of COVID-19 [31]. The largest cohort study available included 1099 COVID-19 cases from China and showed that 22.2% (168/757) of patients exhibited elevated AST and 21.3% (158/741) exhibited elevated ALT. Compared to non-severe patients, severe patients were more likely to have elevated ALT (28.1% vs 19.8%) and elevated AST (39.4% vs 18.2%) [32]. This is consistent with our observation that a large number of patients with the Omicron variant develop abnormal liver function.

In addition to the above-mentioned liver impairment in COVID-19 patients caused by specific serum metabolites, there are many studies suggesting that SARS-CoV-2 infection may cause liver impairment in patients through other mechanisms. For example, Wang *et al.* found that SARS-CoV-2-infected hepatocytes showed significant mitochondrial swelling, reduction of glycogen granules and endoplasmic reticulum expansion, suggesting that SARS-CoV-2 infection in the liver can directly contribute to liver injury in COVID-19 patients [33]. In addition, it has also been shown that immune stress and inflammatory factor storms are also important mechanisms causing liver injury in COVID-19 patients. Elevated levels of inflammatory cytokines (e.g., IL-6 and IL-10) and a decline in CD4 + T cells in patients with COVID-19 liver injury have been determined to be independent risk factors for severe liver injury [34,35]. Further understanding of the mechanisms by which SARS-CoV-2 infection causes liver injury in patients will help us to make more targeted treatments for patients.

We identified a significant reduction in circulating metabolite Prenylcysteine in the serum of patients with the Omicron variant compared to healthy individuals, which may increase the risk of atherosclerosis in patients. A *et al.* found that prenylcysteine can act as the substrate of prenylcysteine oxidase 1 to react with it to generate oxidant (H2O2), which promotes the ability of apoB100-containing lipoproteins to oxidize, and ultimately plays a role in the development of atherosclerotic lesions [36]. Reduced prenylcysteine in patients with the Omicron variant indicates that more atherosclerosis-causing oxidants are produced in their bodies, and therefore they are at higher risk for cardiovascular disease compared to the healthy individuals. A study that enrolled 4,131,717 participants who underwent SARS-CoV-2 testing similarly supports our view. They followed subjects from 30 days to 12 months and showed that compared with non-COVID-19 controls (n = 2249533), COVID-19 survivors (n = 691455) had a significantly higher 12-month risk of incident cardiovascular disease (including inflammatory heart disease, cerebrovascular disease, ischemic heart disease, and other cardiac diseases such as thromboembolic disease and heart failure) [37].

In addition to our findings of impaired urea cycling and elevated risk of developing atherosclerosis in patients with the Omicron variant, Kimhofer *et al.* characterized the effects of SARS-CoV-2 infection on human plasma metabolism through a multiplatform combined phenotype. They found elevated α-1-acid glycoprotein and kynurenine/tryptophan ratios in COVID-19 patients. In addition, they found that the amino acid marker profile of glucose and hepatic dysfunction in COVID-19 patients was consistent with that of patients with coronary artery disease, which is highly consistent with our finding that patients with the Omicron variant are at elevated risk of atherosclerosis and have hepatic dysfunction [38]. In addition, analysis of metabolomics by Bruzzone *et al.* revealed pathogenic redistribution of the size and composition of lipoprotein particles in COVID-19 patients, which increased the risk of atherosclerosis, whereas analysis of metabolomics showed abnormally high levels of ketone bodies and 2-hydroxybutyric acid, an indicator of hepatic glutathione synthesis and a marker of oxidative stress, which suggests that SARS-CoV-2 infection-induced liver injury is associated with oxidative stress and hypolipidemia [39]. Bojkova *et al.* established a culture model of clinically isolated SARS-CoV-2-infected human cells and determined SARS-CoV-2 infection at different times after infection by translational and proteomics, and found that SARS-CoV-2 remodeled central cellular pathways including nucleic acid metabolism and protein homeostasis [40]. Overall, elucidation of the metabolic profiles, lipid profiles, and protein profiles of COVID-19 patients will expand our understanding of disease pathogenesis and provide clues to potential therapeutic strategies.

Our study also showed that although the COVID-19 nucleic acid tests of the recovered cases had been negativing twice consecutively, meeting the guideline criteria for discharge, many of their circulating metabolites had not returned to normal levels. This is consistent with the findings of wang *et al* [31]. Therefore, better care and nutritional support should be provided to Omicron patients even after they have met the guideline criteria for discharge so that they can recover more quickly from infection with the Omicron variant.

We successfully recruited 336 cases of the Omicron variant, but all of these cases were from Henan Province, and additional inclusion of a cross-regional cohort for validation may be necessary to confirm our findings. However, the results of the validation cohort and the discovery cohort were highly consistent, solidifying our results. In addition, due to practical constraints, the vast majority of the samples we collected were from mildly ill or asymptomatic patients and were not stratified for disease severity. We were therefore unable to explore the relationship between metabolites and disease severity. However, as research proceeds, metabolomics will play a unique role in the diagnosis and treatment of disease.

Overall, we provided a comprehensive view of the serum circulating metabolite profiles of Omicron variant and recovered cases and found that Omicron variant cases had an impaired urea cycle and an elevated risk of atherosclerosis. In addition, we developed a non-invasive diagnostic model for Omicron variant patients based on a large clinical cohort for the first time, which had a diagnostic efficacy of 99.7% in the validation cohort.

## Supporting information

S1 Fig Study design and flow diagram. A total of 979 participants were enrolled and provided serum samples in the study. After excluding data with significant biases, 932 serum sample data were ultimately used, including 336 OC, 216 RC, and 380 HC. These data were divided into a discovery cohort for differential metabolite screening and diagnostic modeling, and a validation cohort for verifying the efficacy of the diagnostic model, in a 3:1 ratio.
(DOCX)

S1 Data Metabolome data of the discovery cohort. (XLSX)

S2 Data Metabolome data of the validation cohort.
(XLSX)

S3 Data Metabolites detected in the samples by untargeted metabolomics.
(XLSX)

S4 Data Overview of total changed metabolites.
(XLSX)

S5 Data KEGG enrichment analysis of OC vs HC.
(XLSX)

S6 Data KEGG enrichment analysis of RC vs HC.
(XLSX)

S7 Data Relative intensities of differential metabolites that may serve as potential metabolic biomarkers in Omicron variant cases.
(XLSX)

S8 Data Unary linear regression analysis of the correlation between age and significantly differential metabolites in Omicron variant cases.
(XLSX)

S9 Data Correlation between microbiome, metabolome and clinical indicators in Omicron variant cases.
(XLSX)

## Abbreviations

COVID-19: Corona Virus Disease
OC: omicron variant cases
RC: recovered cases
HC: healthy controls
SARS-CoV-2: severe acute respiratory syndrome coronavirus 2
LC-MS: liquid chromatography-mass spectrometry
OPLS-DA: orthogonal partial least-squares discriminant analysis
QC: quality control
PCA: principal component analysis principal component analysis
VIP: Variable importance in the projection
KEGG: Kyoto Encyclopedia of Genes and Genomes
AUC: area under the curve
