# Supplementary material for: Alterations in the serum metabolome in patients with the COVID-19 Omicron variant and in recovered cases
Source: PLoS One. 2025 Oct 14;20(10):e0327297. doi: 10.1371/journal.pone.0327297 (PMC12520338; doi:10.1371/journal.pone.0327297)
Supplement: S1 Fig — A total of 979 participants were enrolled and provided serum samples in the study. After excluding data with significant biases, 932 serum sample data were ultimately used, including 336 OC, 216 RC, and 380 HC. These data were divided into a discovery cohort for differential metabolite screening and diagnostic modeling, and a validation cohort for verifying the efficacy of the diagnostic model, in a 3:1 ratio. (DOCX) [file pone.0327297.s001.docx]

**Supplementary materials**

**Supplementary method**

**Diagnostic, inclusion, and exclusion criteria**

Diagnostic criteria are based on the “COVID-19 diagnosis and treatment program trial V.9 guidelines” issued by the National Health Commission of the People’s Republic of China.

1. Suspected case

There is any one of the following epidemiological histories and conforms to any two of the clinical manifestations.

If there is no clear history of epidemiology, it conforms to 3 of the clinical manifestations, or conforms to any 2 of the clinical manifestations, and novel coronavirus's specific IgM antibody is positive (those who have recently been vaccinated with novel coronavirus vaccine are not taken as a reference index).

(1) History of Epidemiology

①Travel history or residence history in the community where the case was reported within 14 days prior to onset of illness.

②A history of contact with novel coronavirus infection within 14 days before the onset of the disease.

③Had contact with patients with fever or respiratory symptoms from the reported community within 14 days before the onset of the disease.

④Cluster onset (2 or more cases of fever and / or respiratory symptoms within 14 days in a small area such as home, office, school, class, etc.).

(2) Clinical manifestation

COVID-19-related clinical manifestations such as fever and / or respiratory symptoms.

②With the above-mentioned imaging features of COVID-19.

③In the early stage of the disease, the total leukocyte count was normal or decreased, and the lymphocyte count was normal or decreased.

2. Confirmed case

The suspected case has one of the following etiological or serological evidence:

(1) Novel coronavirus tested positive for nucleic acid.

(2) Novel ①coronavirus specific IgM antibody and IgG antibody were positive in those who were not vaccinated with novel coronavirus vaccine.

3.Rehabilitation case

①The body temperature returned to normal for more than 3 days.

②Respiratory symptoms obviously improved.

③Pulmonary imaging showed that acute exudative lesions were significantly improved.

④The Ct values of N gene and ORF gene detected by novel coronavirus nucleic acid were ≥ 35 for two consecutive times (RT-PCR method, the limit value was 40, sampling time was at least 24 hours), or novel coronavirus nucleic acid test was negative for two consecutive times (RT-PCR method, the cutoff value was less than 35, sampling time was at least 24 hours).

Those who meet the above conditions can be discharged.

All registered persons are examined by a professional stomatologist to ensure the health of the mouth and gums. The samples of all healthy volunteers are from the physical examination Department of the first affiliated Hospital of Zhengzhou University. The inclusion criteria can be referred to our previous study. The exclusion criteria included diabetes, obesity, hypertension, metabolic syndrome, irritable bowel syndrome, non-alcoholic fatty liver disease, liver cirrhosis and celiac disease. Individuals who received antibiotics and / or probiotic treatment within 8 weeks prior to registration were also excluded.

**Enrollment process**

COVID-19 designated hospital in Henan Province has set up a number of suspected case wards and confirmed case wards. The suspected case ward is used to treat patients who meet the conditions of suspected cases in the guidelines for diagnosis and treatment. After admission, professionals collect throat swab samples from related patients for RT-PCR nucleic acid testing. If the nucleic acid is positive, it is immediately transferred to the confirmed case ward. Our investigators will then assess whether the patient meets our registration criteria. If the conditions are met, the patient will be included in the confirmed case group after signing the informed consent. In the end, our investigators screened and recruited eligible patients. The patients who met the conditions of rehabilitation cases in the diagnosis and treatment guidelines were transferred to the out-of-hospital isolation area and observed for 15 days. If the nucleic acid test is not negative during this period, the recovered patient can leave the isolation area. Our investigators will collect samples from them two days before discharge.

**Supplementary figure legend**

**Figure S1. Research design.** A total of 979 serum samples were collected in Henan Province and sequenced for untargeted metabolomics. After screening, we analyzed 336 OC, 216 RC and 380 HC. We finally screened for differential metabolites and established a noninvasive diagnostic model for Omicron. OC, Omicron variant cases; RC, recovered cases; HC, healthy controls.

**Supplementary date**

**Data S1.** Metabolome data of the discovery cohort

**Data S2.** Metabolome data of the validation cohort

**Data S3.** Metabolites detected in the samples by untargeted metabolomics

**Data S4.** Overview of total changed metabolites

**Data S5.** KEGG enrichment analysis of OC vs HC

**Data S6.** KEGG enrichment analysis of RC vs HC

**Data S7.** Relative intensities of differential metabolites that may serve as potential metabolic biomarkers in Omicron variant cases

**Data S8.** Unary linear regression analysis of the correlation between age and significantly differential metabolites in Omicron variant cases

**Data S9.** Correlation between microbiome, metabolome and clinical indicators in Omicron variant cases
